# Modelling impacts of food industry co-regulation on noncommunicable disease mortality, Portugal

**DOI:** 10.2471/BLT.18.220566

**Published:** 2018-05-14

**Authors:** Francisco Goiana-da-Silva, David Cruz-e-Silva, Luke Allen, Maria João Gregório, Milton Severo, Paulo Jorge Nogueira, Alexandre Morais Nunes, Pedro Graça, Carla Lopes, Marisa Miraldo, João Breda, Kremlin Wickramasinghe, Ara Darzi, Fernando Araújo, Bente Mikkelsen

**Affiliations:** a Centre for Health Policy, Institute of Global Health Innovation, Imperial College London, South Kensington Campus, London SW7 2AZ, England.; b Centre for Innovation, Technology and Policy Research, University of Lisbon, Lisbon, Portugal.; c Nuffield Department of Primary Care Health Sciences, University of Oxford, Oxford, England.; d Faculty of Nutrition and Food Sciences, University of Porto, Oporto, Portugal.; e Department of Public Health and Forensic Sciences, and Medical Education, Faculty of Medicine, University of Porto, Oporto, Portugal.; f Preventive and Public Health Institute, Faculty of Medicine, University of Lisbon, Lisbon, Portugal.; g Centre for Public Administration and Public Policies,Institute of Social and Political Sciences, University of Lisbon, Lisbon, Portugal.; h Department of Management & Centre for Health Economics and Policy Innovation, Imperial College Business School, London, England.; i WHO European Office for the Prevention and Control of Noncommunicable Diseases, WHO Regional Office for Europe, Moscow, Russian Federation.; j Department of Surgery and Cancer, Imperial College London, London, England.; k University Hospital of São João, Faculty of Medicine, University of Porto, Oporto, Portugal.; l Division of Noncommunicable Diseases and Promoting Health through the Life-course, WHO Regional Office for Europe, Copenhagen, Denmark.

## Abstract

**Objective:**

To model the reduction in premature deaths attributed to noncommunicable diseases if targets for reformulation of processed food agreed between the Portuguese health ministry and the food industry were met.

**Methods:**

The 2015 co-regulation agreement sets voluntary targets for reducing sugar, salt and trans-fatty acids in a range of products by 2021. We obtained government data on dietary intake in 2015–2016 and on population structure and deaths from four major noncommunicable diseases over 1990–2016. We used the Preventable Risk Integrated ModEl tool to estimate the deaths averted if reformulation targets were met in full. We projected future trends in noncommunicable disease deaths using regression modelling and assessed whether Portugal was on track to reduce baseline premature deaths from noncommunicable diseases in the year 2010 by 25% by 2025, and by 30% before 2030.

**Findings:**

If reformulation targets were met, we projected reductions in intake in 2015–2016 for salt from 7.6 g/day to 7.1 g/day; in total energy from 1911 kcal/day to 1897 kcal/day due to reduced sugar intake; and in total fat (% total energy) from 30.4% to 30.3% due to reduced trans-fat intake. This consumption profile would result in 248 fewer premature noncommunicable disease deaths (95% CI: 178 to 318) in 2016. We projected that full implementation of the industry agreement would reduce the risk of premature death from 11.0% in 2016 to 10.7% by 2021.

**Conclusion:**

The co-regulation agreement could save lives and reduce the risk of premature death in Portugal. Nevertheless, the projected impact on mortality was insufficient to meet international targets.

## Résumé

**Objectif:**

Modéliser la diminution des décès prématurés attribués à des maladies non transmissibles lorsque les objectifs en matière de reformulation des produits alimentaires transformés conjointement fixés par le ministère portugais de la Santé et l'industrie alimentaire sont atteints.

**Méthodes:**

L'accord de 2015 sur la coréglementation définit des objectifs volontaires pour réduire la teneur en sucre, en sel et en acides gras trans de divers produits d'ici à 2021. Nous avons obtenu des données publiques sur l'apport alimentaire en 2015-2016 et sur la structure de la population et les décès dus à quatre maladies non transmissibles majeures entre 1990 et 2016. Nous avons utilisé l'outil « Preventable Risk Integrated ModEl » pour estimer les décès qui pourraient être évités si les objectifs en matière de reformulation étaient pleinement atteints. Nous avons projeté l'évolution future des décès dus à des maladies non transmissibles à l'aide d'une modélisation par régression et déterminé si le Portugal était en bonne voie pour diminuer de 25% d'ici à 2025 et de 30% avant 2030 les décès prématurés dus à des maladies non transmissibles par rapport à l'année de référence, à savoir 2010.

**Résultats:**

Si les objectifs en matière de reformulation étaient atteints, nous avons projeté que la consommation de sel en 2015-2016 passerait de 7,6 g/jour à 7,1 g/jour; l'énergie totale de 1911 kcal/jour à 1897 kcal/jour en raison d'une réduction de la consommation de sucre; et les matières grasses totales (% de l'énergie totale) de 30,4% à 30,3% en raison d'une réduction de la consommation de gras trans. Ce profil de consommation se traduirait par 248 décès prématurés en moins dus à des maladies non transmissibles (IC à 95%: de 178 à 318) en 2016. Nous avons projeté que la pleine mise en œuvre de l'accord avec l'industrie permettrait de faire passer le risque de décès prématuré de 11,0% en 2016 à 10,7% en 2021.

**Conclusion:**

L'accord sur la coréglementation pourrait sauver des vies et réduire le risque de décès prématuré au Portugal. L'impact prévu sur la mortalité était néanmoins insuffisant pour atteindre les objectifs internationaux.****

## Resumen

**Objetivo:**

Modelizar la reducción de muertes prematuras atribuidas a las enfermedades no transmisibles si se cumplen los objetivos de reformulación de los alimentos procesados acordados entre el Ministerio de Salud portugués y la industria alimentaria.

**Métodos:**

El acuerdo de corregulación de 2015 establece objetivos voluntarios para reducir el azúcar, la sal y los ácidos grasos trans en una serie de productos para 2021. Se obtuvieron datos gubernamentales sobre la ingesta alimentaria en 2015-2016 y sobre la estructura de la población y las muertes por cuatro enfermedades no contagiosas principales en el periodo entre 1990 y 2016. Se utilizó la herramienta Preventable Risk Integrated ModEl (Modelo integrado de riesgos evitables) para estimar las muertes que se evitarían si se cumplían plenamente los objetivos de la reformulación. Se proyectaron las tendencias futuras de las muertes por enfermedades no transmisibles utilizando modelos de regresión y se evaluó si Portugal estaba en camino de reducir las muertes prematuras de referencia por enfermedades no contagiosas del año 2010 en un 25 % para 2025 y en un 30 % antes de 2030.

**Resultados:**

Si se cumplieran los objetivos de la reformulación, se proyectó una reducción de la ingesta de sal en 2015-2016 de 7,6 g/día a 7,1 g/día; de la energía total de 1911 kcal/día a 1897 kcal/día debido a la reducción de la ingesta de azúcar; y de grasa total (% de la energía total) del 30,4 % al 30,3 % debido a la reducción de la ingesta de grasas trans. Este perfil de consumo resultaría en 248 muertes prematuras por enfermedades no transmisibles menos (IC del 95 %: 178 a 318) en 2016. Se estimó que la plena aplicación del acuerdo de la industria reduciría el riesgo de muerte prematura del 11,0 % en 2016 al 10,7 % en 2021.

**Conclusión:**

El acuerdo de corregulación podría salvar vidas y reducir el riesgo de muerte prematura en Portugal. No obstante, los efectos previstos sobre la mortalidad son insuficientes para alcanzar las metas internacionales.

## ملخص

**الغرض:**

وضع نموذج لتخفيض الوفيات المبكرة الناتجة عن الأمراض غير المعدية، إذا تمت تلبية أهداف إعادة صياغة الأغذية المصنعة، والمتفق عليها بين وزارة الصحة البرتغالية وقطاع الأغذية.

**الطريقة:**

تضع اتفاقية التنظيم المشترك لعام 2015 أهدافًا تطوعية لخفض السكر والأملاح والأحماض الدهنية المتحولة في مجموعة من المنتجات بحلول عام 2021. حصلنا على بيانات حكومية عن الاستهلاك الغذائي في الفترة 2015 و2016 وعن التركيبة السكانية والوفيات الناجمة عنها أربعة أمراض رئيسية غير معدية خلال الفترة من 1990 إلى 2016. كما استعنا بأداة النموذج المتكامل للمخاطر القابلة للوقاية منها، وذلك لتقييم حالات الوفاة التي يتم تجنبها، إذا ما تم تلبية أهداف إعادة الصياغة بشكل كامل. قمنا بوضع توقعات للاتجاهات المستقبلية لوفيات الأمراض غير المعدية باستخدام نماذج موضوعة للتحوف، كما قمنا بتقييم ما إذا كانت البرتغال تسير على الطريق الصحيح للحد من الوفيات المبكرة الأساسية الناجمة عن الأمراض غير المعدية في عام 2010 بنسبة 25% بحلول عام 2025، وبنسبة 30% قبل عام 2030.

**النتائج:**

لقد توقعنا أنه إذا تم تلبية أهداف إعادة الصياغة، سيكون هناك انخفاض في الاستهلاك في عامي 2015 و2016 من الملح من 7.6 جم/يوم إلى 7.1 جم/يوم؛ وفي إجمالي الطاقة من 1911 كيلو كالوري/يوم إلى 1897 كيلو كالوري/يوم، وذلك نتيجة لانخفاض استهلاك السكر؛ وفي إجمالي الدهون (النسبة المئوية لإجمالي الطاقة) من 30.4% إلى 30.3% بسبب انخفاض استهلاك الدهون المتحولة. سيؤدي هذا النمط من الاستهلاك إلى انخفاض في حالات الوفيات المبكرة الناجمة عن الأمراض غير المعدية بمعدل 248 حالة أقل (فاصل الثقة 95%: 178 إلى 318) في عام 2016. لقد توقعنا أن التنفيذ الكامل لاتفاقية القطاع سوف تحد من خطر الوفاة المبكرة من 11.0% في عام 2016، إلى 10.7% بحلول عام 2021.

**الاستنتاج:**

يمكن أن تؤدي اتفاقية التنظيم المشترك لإنقاذ الحياة، والحد من خطر الوفيات المبكرة في البرتغال. ورغم ذلك، فإن التأثير المتوقع على معدل الوفيات كان غير كافٍ لتلبية الأهداف الدولية.

## 摘要

**目的:**

如果葡萄牙卫生部及其食品工业间达成协议，对加工食品进行重新配方，则其将影响非传染性疾病引起的过早死亡率。我们就此下降趋势进行建模。

**方法:**

2015 年的共同监管协议规定，到 2021 年，主动减少一系列产品中的糖、盐和反式脂肪酸。我们获得了 2015–2016 年膳食摄入以及 1990–2016 年四种主要非传染性疾病相关人口结构和死亡情况的政府数据。我们采用可预防风险综合模型工具，估计出若完全达到重新配方目标可避免死亡的人数。我们采用回归模型，预测了非传染性疾病死亡的未来趋势，并评估了葡萄牙是否有望在 2010 年将非传染性疾病基线过早死亡率降低 20％，到 2025 年降低 25％，到 2030 年降低 30％。

**结果:**

如果重新配方得以实现，我们预计 2015–2016 年盐的摄入量将从 7.6 克/天减少至 7.1 克/天；由于糖摄入量的减少，总能量从 1911 千卡/天减少至 1897 千卡/天；由于反式脂肪摄入量的减少，总脂肪（％ 总能量）从 30.4％ 减少至 30.3％。此类消耗情况将导致 2016 年非传染性疾病过早死亡人数减少 248 人（95％ 置信区间，CI：178 至 318人）。我们预计行业协议的全面实施将令过早死亡风险从 2016 年的 11.0％ 降至 2021 年的 10.7％。

**结论:**

共同监管协议可挽救生命并降低葡萄牙过早死亡的风险。然而，对死亡率的预计影响仍未达到国际目标。****

## Резюме

**Цель:**

Создание модели уменьшения показателей преждевременной смертности, связанной с неинфекционными заболеваниями, если будут достигнуты согласованные Министерством здравоохранения Португалии и представителями пищевой промышленности цели в области изменения рецептур технологически переработанных продуктов питания.

**Методы:**

Заключенное в 2015 году соглашение о совместном регулировании задает добровольные цели в части уменьшения содержания сахара, соли и трансжирных кислот в ряде продукции к 2021 году. Авторы получили данные о пищевом рационе населения за 2015–2016 годы и данные о составе населения и смертности от четырех основных неинфекционных заболеваний за период с 1990 по 2016 год, предоставленные государственными учреждениями. Применялся метод интегрированного моделирования предотвращаемого риска (Preventable Risk Integrated ModEl) для оценки количества смертей, которые можно было бы предотвратить, если бы цели в области изменения состава продукции были полностью достигнуты. Авторы спрогнозировали будущие тенденции изменения показателей смертности от неинфекционных заболеваний при помощи регрессионного моделирования и оценили, смогут ли власти Португалии достичь уменьшения исходного показателя преждевременной смертности от неинфекционных заболеваний (принимая за базовое значение уровень 2010 года) на 25% к 2025 году и на 30% до 2030 года.

**Результаты:**

Исходя из предположения о достижении поставленных целей, авторы спрогнозировали снижение потребления соли в 2015–2016 годах с 7,6 до 7,1 г/день, снижение общей калорийности пищи с 1911 до 1897 ккал/день в связи со снижением потребления сахара, а также общего потребления жиров (в % от общей калорийности) с 30,4 до 30,3% вследствие уменьшения потребления трансжиров. Такой профиль потребления может привести к снижению показателя преждевременных смертей от неинфекционных заболеваний на 248 случаев (95%-й ДИ: 178–318) в 2016 г. Авторы прогнозируют, что полноценное осуществление отраслевых соглашений может снизить риск преждевременной смертности с 11,0% в 2016 году до 10,7% в 2021 году.

**Вывод:**

Соглашение о совместном регулировании может спасти жизни людей и уменьшить риск преждевременной смерти для жителей Португалии. Однако прогнозируемое влияние на смертность недостаточно для того, чтобы соответствовать международным целевым значениям.

## Introduction

In 2017, 88% (96 587) of 109 758 deaths in the Portuguese population of 10 291 027 were attributed to noncommunicable diseases.^1^ Portugal has committed to the United Nations sustainable development goal (SDG) target 3.4 to reduce premature mortality from noncommunicable diseases by one third by 2030 and the voluntary target to reduce these deaths by one quarter by 2025 from the baseline year 2010. To date, there have been no efforts to evaluate Portugal’s performance against these targets.^2^^–^^4^

Dietary risk factors are the leading preventable cause of noncommunicable diseases morbidity and mortality in Portugal.^1^^,^^2^ In response to the increasing prevalence of noncommunicable diseases the government introduced the National Programme for the Promotion of Healthy Eating in 2012.^5^^,^^6^ According to national data, the mean daily intake of free sugars in 2015–2016 was 35 g/day and about 24% (2 600 00) of the population exceeded the World Health Organization (WHO) recommended limits for free-sugar consumption. Non-adherence to this recommendation was more prevalent among children (48.4%; 380 000) and adolescents (48.7%; 422 000). For salt intake, 76.4% (8 283 000) of the population exceed the WHO recommended upper limits for daily sodium consumption. Encouragingly, trans-fatty acids (TFAs) intake constituted more than 1% of the total energy intake for only 0.4% (43 000) of the population.^4^

In 2017, Portugal introduced a consumption tax on sugar-sweetened beverages. The tax was set at euro (€) 8.22 per hectolitre of finished product for drinks with < 80 g sugar/L, and €16.46 for finished products with > 80 g/L sugar.^7^ Preliminary results from the first year of the tax implementation in 2017 showed that the mean energy content of sugar-sweetened beverages fell by 11% (from 30.92 kcal per 100 mL to 27.45 kcal per 100 mL). Sales of these drinks have decreased by almost 7% (from 538 million litres in 2016 to 503 million litres in 2017).^7^ Inspired by the success of the tax,^8^ the government proposed a salt tax to be levied on processed foods. The Portuguese parliament rejected this proposal and recommended instead introducing a co-regulation agreement with the food industry, whereby the government defines food reformulation targets and agrees a follow-up and accountability process with industry. If the targets are not met, stronger measures to promote reformulation, such as taxation, shall be implemented by the government. Such agreements have been adopted by several other countries.^9^^–^^15^

The agreement, drafted by the Portuguese health ministry, included reformulation targets and public accountability guidelines on all processed foods high in salt, sugar and TFAs.^16^ Guidelines were based on the recommendations of the European Commission’s High-Level Group on Nutrition and Physical Activity^17^^–^^19^ and an analysis of the consumption patterns of the population.^20^^,^^21^ A consensus was reached among different stakeholders (the Portuguese nutrition association, nutritionist college and consumer protection association) on several food categories that should be reformulated. Defining the targets for the year 2021 would follow a baseline assessment in December 2017 of the nutritional content of processed food products representing at least 80% of the market share. Building on a consultation with the relevant experts, researchers, health professionals and representatives from the food and health sectors, the health ministry established annual milestones as well as a final reformulation target for each food sector (Table 1). The values were derived from the experience of the United Kingdom of Great Britain and Northern Ireland and recommendations from the High Level Group on Nutrition and Physical Activity of the European Commission^11^^,^^22^ and the World Health Organization (WHO).^23^ The reduction targets were 16% for salt, 20% for sugar and a limit of 2 g TFAs per 100 g of fat in margarines and shortening by 2021. The Portuguese government proposed additional targets for reducing salt in bread by 30% by 2021, corresponding to a maximum level of 1 g salt per 100 g of bread, and a limit of 1 g TFAs per 100 g of fat in pastry, by 2021.

**Table 1:** Preliminary objectives of the 2015 co-regulation agreement between the Portuguese government and the food industry for reducing sugar, salt and trans-fatty acids in processed food

Nutrient	Food products to reformulate	Nutrient reduction target by year			Total reduction by 2021
		2019	2020	2021	
Sugar	Breakfast cereals; cookies and biscuits; chocolate milk; yogurt; soft drinks; fruit juice	5%	7%	8%	20%
Salt	Bread (toast); breakfast cereals; cheese; cookies and biscuits; potato chips and other snacks; processed meats (ham); ready-to-eat soups	4%	5%	7%	16%
	Bread	10% (1.2 g salt per 100 g bread)	10% (1.1 g salt per 100 g bread)	10% (1.0 g salt per 100 g bread)	30%
Trans-fatty acids	Cookies and biscuits; fat spreads	< 2 g trans fatty acids per 100 g of fat			
	Pastries	< 2 g trans fatty acids per 100 g of fat		< 1 g trans fatty acids per 100 g of fat	

Note: All percentage reductions are based on baseline levels from December 2017.

We modelled the reduction in premature mortality associated with noncommunicable diseases that would be expected if the Portuguese government’s co-regulation agreement with the food industry were established and the preliminary targets for food reformulation were met in full. We also aimed to analyse whether Portugal is on track to meet SDG targets to reduce premature mortality from noncommunicable diseases by 25% by 2025 and 33% by 2030.

## Methods

### Study design

In this modelling study carried out in May 2018, we used data on dietary intake, noncommunicable disease mortality and demographic data from 2016 to project how many lives could be saved in the same year if the industry co-agreement targets were met in full. We also modelled the trends in mortality from noncommunicable diseases in Portugal from 1990 to 2030 and what impact food reformulation would have on mortality trends.

### Data sources

We used population data on food consumption for those aged 15–84 years, obtained from the Portuguese National Food, Nutrition and Physical Activity Survey conducted from October 2015 to September 2016.^4^ The survey collected nationwide and regional data on dietary habits, physical activity and anthropometrics from a representative sample of the Portuguese general population aged between 3 months and 84 years.^24^ Participants were selected from the national health registry by multistage sampling and 5811 individuals completed food consumption interviews, assessed by 24-hour recall.^25^

The Portuguese directorate general of health provided data for the years 1990–2016 on the age (5-year age bands) and sex distribution of the population and the annual numbers of deaths attributed to four major noncommunicable diseases. The ministry codes deaths attributed to noncommunicable diseases using the International Statistical Classification of Diseases and Related Health Problems, 10th revision (ICD-10), as follows: circulatory system diseases (codes I00–I99); diabetes (E10–E14); malignant neoplasms (C00–C97); and chronic respiratory diseases (J30–J98).

### Data analysis

#### Modelling changes in nutrient consumption 

We used the Electronic Assessment Tool for 24 hours recall (eAT24) software^25^ to convert food consumption data to intake of total energy (kcal/day), sodium (g/day) and fat (% of total fat/day). We then used the Statistical Program to Assess Dietary Exposure software (Dutch National Institute for Public Health and the Environment, Bilthoven, Netherlands) to estimate the population’s usual intake of nutrients, removing intra-individual variability.^26^

We used the following formula to calculate the projected daily dietary intakes of nutrients if the co-regulation agreement targets were achieved in full. The formula combined consumption data from the 2015–2016 national survey with the nutrient concentration in the industry co-regulation agreement (*c_f_* ):
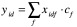
(1)where *y_id_* corresponds to the total energy, sodium or percentage of fat of individual *i* at day *d* and* x_idf_* corresponds the consumption of individual *i* at day *d* of food item *f*. Taking the 2015–2016 levels of consumption of each food as the baseline, we predicted the intakes of total energy, sodium and fat by the population using the updated concentrations of nutrients after reformulation.

#### Modelling changes in premature mortality

To model reductions in premature noncommunicable disease deaths (that is deaths at ages 30–69 years), we used the Preventable Risk Integrated ModEl, Nov. 2017 version, an openly available statistical noncommunicable disease modelling tool.^27^ The tool is currently being adopted by WHO Europe to help Member States estimate the impacts of changes in nutrition policy and hence prioritize noncommunicable disease policy options. Users input baseline data on mortality rates, population structure and behavioural risk factors (in this case, nutritional intake), along with a counterfactual scenario. The model predicts changes in mortality for any of 24 noncommunicable diseases, based on findings from international meta-analyses.^27^ As the modelling tool is cross-sectional, we could only compare a historical scenario (the population mortality rates and dietary consumption in 2016) with a counterfactual scenario (where the population’s consumption of salt, sugar and TFA was reduced) and calculate the expected number of deaths that would be observed in the same year.

The output of our analysis was the number of deaths that would have been averted in 2016 had the industry co-regulation targets been met in full. In line with the targets listed in Table 1, we modelled population sugar consumption reduced by 20%, salt consumption reduced by 16% (30% for bread), and complete elimination of trans-fats (which corresponds to achieving the targets previously identified). To model the impact of trans-fat reduction we calculated the percentage of total energy constituted by total fats. We reduced the calorie content of the sugar-related foods in Table 1 by 20% to model the impact of sugar reduction. We used Monte Carlo simulation to generate 95% confidence intervals (CI) around point estimates of numbers of deaths averted. The Monte Carlo analysis used uncertainty parameters based on the associations between dietary risk factors and disease outcomes, as described in the literature.^27^

To model the change in risk of premature noncommunicable disease mortality from 1990–2016, we used linear projections to forecast future probability of death. These projections were based on estimates from weighted and non-weighted exponential and linear regressions models to project premature noncommunicable disease mortality to 2030. Weights were exponentially distributed and calculated to be inverse to time (i.e. more recent data was given a heavier weighting than older data). We optimized the weights to have minimum distance between the projections and the two most recent data points. To assess how the co-regulation agreement would impact Portugal’s trajectory we re-ran the projection with the reduced noncommunicable disease deaths that were calculated by the Preventable Risk Integrated ModEl.

## Results

Table 2 presents baseline data for mean intakes of salt, energy and fat derived from the 2016 Portuguese National Food, Nutrition and Physical Activity Survey, along with the projected values for 2016 if the food reformulation targets were met in full. We predicted reductions in mean intakes of salt from 7.6 g/day (standard deviation, SD: 2.3) to 7.1 g/day (SD: 2.2); total energy from 1911 kcal/day to 1897 kcal/day; and total fat as a percentage of total energy per day from 30.4% (SD: 4.8) to 30.3% (SD: 4.8). 

**Table 2:** Projected daily intake of salt, total energy and total fat by age and sex in Portugal in 2021 if a co-regulation agreement on the nutrient content of processed food were implemented

Age and sex	Sample, no.	Population, no.	Mean (SD) salt intake, g/day			Mean^a^ total energy intake, kcal/day			Mean (SD) total fat intake, % total energy/day	
			Baseline	Projected		Baseline	Projected		Baseline	Projected
**Total**	4 067	9 494 698	7.6 (2.3)	7.1 (2.2)		1 911	1 897		30.4 (4.8)	30.3 (4.8)
**Male, years**										
15–19	152	292 936	8.5 (2.4)	8.0 (2.2)		2 355	2 325		30.2 (4.4)	29.8 (4.4)
20–24	125	413 473	8.9 (2.4)	8.3 (2.3)		2 429	2 400		30.0 (4.4)	29.8 (4.4)
25–29	115	258 286	9.1 (2.5)	8.5 (2.3)		2 459	2 431		29.8 (4.4)	29.7 (4.4)
30–34	139	321 783	9.2 (2.5)	8.6 (2.4)		2 456	2 430		29.7 (4.4)	29.6 (4.4)
35–39	157	338 404	9.3 (2.5)	8.7 (2.4)		2 423	2 401		29.4 (4.4)	29.4 (4.4)
40–44	187	503 264	9.3 (2.5)	8.7 (2.4)		2 380	2 360		29.1 (4.4)	29.2 (4.4)
45–49	155	379 035	9.3 (2.5)	8.6 (2.4)		2 331	2 313		28.9 (4.4)	29.0 (4.4)
50–54	166	446 686	9.1 (2.5)	8.5 (2.3)		2 258	2 243		28.5 (4.4)	28.6 (4.4)
55–59	173	403 022	9.0 (2.4)	8.3 (2.3)		2 185	2 174		28.1 (4.4)	28.3 (4.4)
60–64	154	396 197	8.7 (2.4)	8.1 (2.3)		2 114	2 106		27.8 (4.4)	27.8 (4.4)
65–69	169	390 285	8.4 (2.4)	7.8 (2.2)		2 027	2 021		27.3 (4.4)	27.3 (4.3)
70–74	93	188 581	8.1 (2.4)	7.5 (2.1)		1 952	1 949		26.9 (4.4)	26.8 (4.3)
75–79	80	145 870	7.7 (2.2)	7.0 (2.0)		1 859	1 859		26.4 (4.3)	26.0 (4.3)
80–84	50	119 865	7.2 (2.1)	6.6 (2.0)		1 775	1 777		25.9 (4.3)	25.3 (4.3)
85+^b^	NA	NA	7.2 (2.1)	6.6 (2.0)		1 775	1 777		25.9 (4.3)	25.3 (4.3)
All ages	1915	4 597 687	8.9 (2.5)	8.2 (2.3)		2 241	2 223		28.8 (4.5)	28.7 (4.5)
**Female, years**										
15–19	183	270 998	6.8 (1.8)	6.4 (1.7)		1 803	1 784		31.8 (4.9)	31.8 (4.9)
20–24	147	348 323	6.8 (1.8)	6.4 (1.7)		1 774	1 756		32.0 (5.0)	31.9 (4.9)
25–29	143	278 977	6.7 (1.8)	6.4 (1.7)		1 741	1 725		32.0 (5.0)	31.9 (4.9)
30–34	182	368 473	6.6 (1.8)	6.3 (1.7)		1 705	1 690		31.9 (4.9)	31.8 (4.9)
35–39	195	434 452	6.6 (1.8)	6.2 (1.7)		1 674	1 660		31.8 (5.0)	31.7 (4.9)
40–44	248	574 407	6.5 (1.8)	6.1 (1.7)		1 643	1 631		31.6 (5.0)	31.5 (4.9)
45–49	190	422 300	6.4 (1.7)	6.0 (1.6)		1 612	1 601		31.3 (4.9)	31.3 (4.9)
50–54	204	493 009	6.4 (1.7)	5.9 (1.6)		1 586	1 576		31.1 (4.9)	31.0 (4.9)
55–59	172	343 994	6.3 (1.7)	5.8 (1.6)		1 558	1 549		30.7 (4.9)	30.7 (4.9)
60–64	130	293 724	6.2 (1.7)	5.7 (1.6)		1 534	1 527		30.4 (4.9)	30.3 (4.9)
65–69	142	481 403	6.2 (1.7)	5.6 (1.6)		1 509	1 502		29.9 (4.9)	29.9 (4.9)
70–74	105	259 438	6.1 (1.6)	5.5 (1.5)		1 489	1 483		29.5 (4.9)	29.5 (4.9)
75–79	73	193 218	6.0 (1.7)	5.4 (1.5)		1 469	1 463		29.1 (4.8)	29.1 (4.9)
80–84	38	134 295	6.0 (1.6)	5.3 (1.5)		1 447	1 442		28.5 (4.9)	28.6 (4.8)
85+^b^	NA	NA	6.0 (1.6)	5.3 (1.5)		1 447	1 442		28.5 (4.9)	28.6 (4.8)
All ages	2 152	4 897 011	6.4 (1.8)	6.0 (1.7)		1 636	1 623		31.1 (5.0)	31.0 (5.0)

NA: not applicable; SD: standard deviation.

^a^ The model which we used to estimate deaths averted due to food reformation does not use SD of mean total energy intake in the calculations.

^b^ For the age group 85+ years we used the same estimates from the previous age group (80–84 years) as the Portuguese National Food, Nutrition and Physical Activity survey only included the population up to 84 years of age.

Notes: The proposed co-regulation agreement between the Portuguese health ministry and the food industry sets targets of reducing sugar by 20%, salt content by 16% (30% for bread) and < 2 g trans-fatty acids per 100 g of fat in a range of products by 2021. The projected (counterfactual) values assumed that the co-regulation targets set by the ministry were fully met. We weighted dietary estimates according to the complex sampling design, considering stratification by the seven Portuguese geographical regions and cluster effect for the selected primary health-care units.^25^

Sources: We obtained baseline data on dietary consumption (24-hour recall) from the Portuguese National Food, Nutrition and Physical Activity Survey in 2015–2016.^24^ Data on age and sex distribution of the population were provided by the Portuguese directorate general of health.

Table 3 shows the projected mean number of noncommunicable disease deaths averted in Portugal in 2016 if targets for reduction of sugar, salt and trans-fats intake by the population were achieved, by age, sex, disease and risk factor. Most of deaths averted would occur in those older than 75 years. Reductions in cardiovascular deaths would greatly outnumber deaths averted from cancer or diabetes. 

**Table 3:** Projected mean number of noncommunicable disease deaths averted in Portugal in 2016 if targets for reduction of sugar, salt and trans-fats intake by the population were achieved, by age, sex, disease and behavioural risk factor

Variable	Population aged > 15 years, no.		No. of deaths averted or delayed		
			2.5th percentile	Mean	97.5th percentile
**Total**	8 873 828		494	800	1106
**By age and sex**					
Age < 75 years	7 819 807		178	248	318
Males	4 148 778		138	272	409
Females	4 725 050		355	527	701
Males aged < 75 years	3 746 359		114	164	215
Females aged < 75 years	4 073 449		63	84	104
**By disease**					
All cardiovascular disease	8 873 828		384	693	999
Coronary heart disease	8 873 828		92	156	221
Stroke	8 873 828		123	233	341
Heart failure	8 873 828		82	144	210
Aortic aneurysm	8 873 828		3	7	11
Pulmonary embolism	8 873 828		2	6	13
Rheumatic heart disease	8 873 828		0	1	3
Hypertensive disease	8 873 828		77	145	213
Diabetes	8 873 828		40	57	70
Chronic obstructive pulmonary disease	8 873 828		0	0	0
Cancer	8 873 828		18	24	30
**By risk factor**					
Diet (excluding obesity)	8 873 828		224	530	840
Diet (including obesity)	8 873 828		494	800	1106
Fruit and vegetables	8 873 828		0	0	0
Fibre	8 873 828		0	0	0
Fats	8 873 828		−4	−1	2
Salt	8 873 828		224	531	841
Physical activity (excluding obesity)	8 873 828		0	0	0
Physical activity (including obesity)	8 873 828		239	274	305
Obesity	8 873 828		239	274	305
Alcohol consumption	8 873 828		0	0	0
Smoking	8 873 828		0	0	0

Notes: The proposed co-regulation agreement between the Portuguese health ministry and the food industry sets targets of reducing sugar by 20%, salt content by 16% (30% for bread) and < 2 g trans-fatty acids per 100 g of fat in a range of products by 2021. We modelled the reduction in premature mortality attributed to noncommunicable diseases that would be observed if the co-regulation targets set by the health ministry were fully met. The population of Portugal in 2016 was 10 309 537. We included all individuals older than 15 years (8 873 828 people). The results were obtained from the Monte Carlo analysis (10 000 simulations).

Sources: We obtained baseline data on dietary habits (24-hour recall) from the Portuguese National Food, Nutrition and Physical Activity Survey in 2015–2016.^24^ Baseline data on mortality and the age and sex distribution of the population were provided by the Portuguese directorate general of health.

We estimated that the reductions in nutrient intakes, if the food reformulation targets were met in full, would avert a total of 798 deaths (95% CI: 483 to 1107) attributed to noncommunicable diseases in 2016 (Table 4). The greatest reduction was for cardiovascular disease, accounting for 692 deaths averted (95% CI: 377 to 999). Achieving the industry targets for food reformulation would avert more deaths among women (526; 95% CI: 348 to 698) than men (272; 95% CI: 132 to 409). Reduction in salt intake made the biggest contribution, accounting for 610 deaths averted (95% CI: 215 to 840) compared with 261 deaths averted (95% CI: 238 to 305) due to reduction of sugar intake and none due to elimination of trans-fat. Of the total noncommunicable disease deaths averted in 2016, 248 (95% CI: 178‒318) were premature deaths.

**Table 4:** Projected number of noncommunicable disease deaths averted in Portugal in 2016 if targets for reduction of sugar, salt and trans-fats intake by the population were achieved, by sex, disease and nutrient

Variable	No. of deaths						
	All deaths attributed to noncommunicable diseases^a^				Premature deaths attributed to noncommunicable diseases^b^		
	Baseline	Projected	Averted (95% CI)		Baseline	Projected	Averted (95% CI)
**Total**	54 745	53 947	798 (483 to 1 107)		17 633	17 386	248 (178 to 318)
**By sex**							
Male	27 699	27 427	272 (132 to 409)		11 744	11 580	164 (113 to 214)
Female	26 424	25 898	526 (348 to 698)		5 899	5 815	84 (63 to 104)
**By disease**							
Cardiovascular disease	11 732	11 040	692 (377 to 999)		2 085	1 899	186 (117 to 256)
Diabetes	4 280	4 219	61 (40 to 71)		944	920	24 (19 to 29)
Chronic obstructive pulmonary disease	2 789	2 789	0 (0 to 0)		518	518	0 (0 to 0)
Cancer	2 335	2 310	25 (18 to 31)		1 162	1 147	15 (10 to 19)
**By nutrient^c^**							
Salt reduction	NA	NA	610 (215 to 840)		NA	NA	NA
Sugar reduction	NA	NA	261 (238 to 305)		NA	NA	NA
Trans-fatty acid elimination	NA	NA	0 (0 to 0)		NA	NA	NA

CI: confidence interval; NA: not applicable.

^a^ We modelled deaths due to four major noncommunicable diseases: circulatory system diseases, diabetes, malignant neoplasms and chronic respiratory diseases.

^b^ Premature deaths were those occurring in 30–69 year olds.

^c^ We estimated deaths related to sugar and trans-fats using change in energy intake. Due to the design of the Preventable Risk Integrated ModEl tool we were unable to obtain estimates of the total number of baseline or counterfactual deaths attributable to the individual nutrients.

Notes: The proposed co-regulation agreement between the Portuguese health ministry and the food industry sets targets of reducing sugar by 20%, salt content by 16% (30% for bread) and < 2 g trans-fatty acids per 100 g of fat in a range of products by 2021. We modelled the reduction in premature mortality attributed to noncommunicable diseases that would be observed if the co-regulation targets set by the health ministry were fully met. The population of Portugal in 2016 was 10 309 537. We included all individuals older than 15 years (8 873 828 people).

Sources: We obtained baseline data on dietary habits (24-hour recall) from the Portuguese National Food, Nutrition and Physical Activity Survey in 2015–2016.^24^ Baseline data on mortality and the age and sex distribution of the population were provided by the Portuguese directorate general of health.

**Fig. 1:**
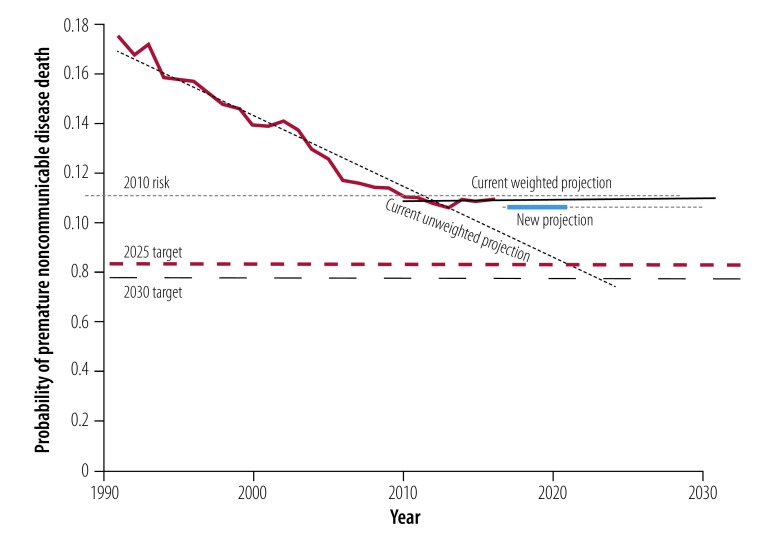
Historic and projected risk of premature noncommunicable disease deaths in Portugal compared with sustainable development goal targets for 2025 and 2030

Notes: The chart line shows the actual data for probability of premature noncommunicable disease mortality from 1990 to 2016 (latest available data). The 2010 risk is the baseline from which the 2025 and 2030 targets are calculated. The unweighted regression line suggests that Portugal will meet both targets before 2025. However, the weighted projection that accords more value to recent data suggests that the risk of premature mortality has stopped declining and will increase towards the 2010 level over the coming 15 years. If the industry co-regulation targets were met in full in the year 2016 then the risk would be lower but, even with these reductions in deaths, the trend will still not reach the 2025 or the 2030 targets.

Source: We obtained baseline data on all-cause mortality and mortality from major noncommunicable diseases (circulatory system diseases, diabetes, malignant neoplasms and chronic respiratory diseases) and the age and sex distribution of the population from the Portuguese directorate general of health. We modelled the change in risk of premature noncommunicable disease mortality from 1990–2016 using linear regression, making projections for the upcoming years. Using the number of estimated averted deaths using the Preventable Risk Integrated ModEl tool, the current population structure (2017), and mortality distribution per causes of deaths and 5-year age groups (2016). We estimated the final unconditional probability of death for 2021 using the life-table method.^43^

Fig. 1 shows that the risk of premature noncommunicable disease death fell from nearly 17.5% to 11.1% between 1990–2010, but remained at 11.1% up to 2016. The SDG target of reducing deaths is 8.3% by 2025 and 7.8% by 2030. The weighted projection estimates a risk (or probability) of death of 11.0% for both 2025 and 2030 based on current trends. Our model shows that the 248 averted premature deaths achieved by fully meeting the food reformulation targets in 2016 would reduce the risk of death to 10.7%. Neither the current weighted trend nor the new projection (assuming that industry targets were met) was set to meet the SDG targets for years 2025 or 2030. The unweighted projection line was the only one crossing the thresholds by the agreed deadlines. WHO data from recent years^28^ suggested that there would be no further reduction in the risk of premature noncommunicable disease mortality given current trends.

## Discussion

Our model predicted that eliminating trans-fats and reducing salt and sugar consumption in the Portuguese population, in line with the food industry co-agreement targets, would have averted 798 deaths due to noncommunicable diseases in 2016, of which 248 were premature. These deaths averted are not sufficient to significantly alter the trends in premature mortality in Portugal or achieve the 2025 and 2030 SDG targets for reduction of premature noncommunicable disease mortality. The Global Burden of Diseases project of the Institute for Health Metrics and Evaluation also publishes mortality projections,^29^ considering three scenarios (reference, better and worse), although not exactly for the same age groups as ours. Their projected worse scenario is similar to our current weighted linear regression projection, while the reference and better scenarios both lie between our weighted and unweighted linear regression projections. Global Burden of Diseases forecast scenarios do not vary much up to 2020 and it is only after that point in time that there is a marked difference in scenarios. How the Institute generates their better scenario is unclear, but the interventions we analysed (reformulation targets for sugar, salt and trans-fats) may be just one of many potential public health interventions being implemented that can help move current trends to the best scenarios.

Several countries have published the results of voluntary industry agreements to promote food reformulation.^9^^–^^15^ The majority of these studies evaluated the impact of reformulation on nutrient intake rather than on health outcomes. An evaluation of the Australian Food and Health Dialogue targets showed modest reductions in the sodium content of bread (9%; from 454 to 415 mg/100 g), breakfast cereals (25%; from 316 to 237 mg/100 g) and processed meats (8%; from 1215 to 1114 mg/100 g) between 2010 and 2013.^14^ Evaluation studies in the United Kingdom also showed that these strategies might be effective in achieving important reductions in the salt content of food. Reductions of 57% (from 0.95 to 0.41 g/100 g) and of 25% (from 0.77 to 0.58 g/100 g) in the salt content of breakfast cereals and sweet biscuits, respectively, were observed between 2004 and 2011.^10^

In terms of health outcomes, interventions focused on salt reduction in food tend to perform favourably.^15^^,^^30^^–^^33^ Estimation of the potential health gains of the Australian food reformulation programme to reduce the salt content in processed foods, implemented since 1989, has shown the potential to avert a total of 5300 disability-adjusted life-years.^20^ A modelling study using data from the Framingham Heart Study in the United States of America suggested that a food reformulation programme to reduce sodium intake by 9.5% could increase quality-adjusted life-years by 2.1 million over current adult lifetimes.^34^ An Argentinian modelling study suggested that reducing sodium in processed meats, cheese and dairy products, soups, cereals, cookies, pizza and pasta by 5–15% could avert 19 000 deaths from all causes over a decade.^35^ Researchers have argued that packaged foods are the priority categories for salt reformulation.^30^ These findings are aligned with previous work suggesting that reformulation can achieve health gains. However, data from previous modelling studies suggests that mandatory approaches generate more health gain than voluntary agreements.^20^^,^^30^^,^^32^ A study in Australia has estimated that health gains from mandatory measures could be 20 times higher than voluntary interventions.^20^

Despite the importance of these data for implementation of healthy eating policies, our study is not without limitations. First, to generate weighted trend lines we used the same statistical approach that the Portuguese health ministry uses for routinely assessing mortality projections. However, the formula heavily discounts older data. As such, the weighted projection may have been overly-pessimistic. Nevertheless. this approach is the national standard that has been used in other national plans and publications.^36^^–^^38^ There is a risk that the slowing rate of decline in noncommunicable disease deaths is an artefact. However, there are several reasons to believe that the rate of decline is slowing, due to stalled improvements in cancer and cardiovascular disease mortality^39^ and the impact of the Portuguese financial crisis in 2011–2014.

Second, the Preventable Risk Integrated ModEl is a cross-sectional model and its strengths and weaknesses are well documented.^27^ Our study fails to reflect major reductions in morbidity associated with reduced consumption of salt, sugar and trans-fats. For example, sugar reductions would impact childhood obesity or diabetes, but these gains were not captured in the analysis. Due to very low population intakes of trans-fats in Portugal,^24^ complete elimination of trans-fats in processed foods did not avert any deaths in our model. It is possible that deaths may have been averted, but that the modelling tool we used did not capture them. Our study only examined the impact of reformulation on mortality from the four major noncommunicable diseases and will therefore underestimate the true reduction in deaths. We also failed to capture reductions in noncommunicable disease morbidity and mortality that extend beyond 2030. These issues mean that the model underestimates the true population health impact. The modelling tool is not designed to directly model the impact of trans-fat changes, except through the changed in percentage of total energy from total fat. Again, this will lead to underestimates of deaths averted. 

The biggest limitation of the modelling tool we used is that it provides an estimate of the number of deaths that would have been averted if the targets had been fully realized in one year (2016 in our study), rather than projecting how many lives would have been saved over the period of roll-out. It is likely that 248 premature deaths would be averted in every year where consumption of salt, sugar and trans-fats were reduced in line with the co-regulation targets, all other things being constant.

Finally, some of the baseline parameters used in this study are likely to underestimate true levels of consumption. We used data from the most recent national dietary survey using self-reported assessment. However, the gold standard for salt assessment is 24-hour urine excretion, as food consumption questionnaires tend to underestimate salt intake. To get a more accurate picture we recommend that the Portuguese health ministry uses 24-hour urine excretion values for monitoring and evaluation of the outcomes of the food reformulation agreement.

### Policy implications

The Portuguese health ministry had attempted a salt-related co-regulation agreement in the past. However, due to the lack of objective evaluation tools there was no appropriate follow-up and therefore no evidence that effective reformulation of processed foods had taken place.^40^ Our model suggests that fully meeting the reformulation targets could avert approximately 250 deaths per year. This figure underestimates the true number of diet-related deaths that would be averted and does not capture the morbidity averted from dietary improvements. Due to their limited impact on premature mortality, we suggest that co-regulation agreements should form part of a broader package of diet policies. These policies might include food labelling, improving the public’s health literacy and environmental interventions, such as health-related food taxes, all of which have been shown to be more equitable and cost–effective than micro-level interventions.^18^^,^^41^^,^^42^

Portugal is currently not on track to reduce premature noncommunicable disease mortality by a quarter by 2025 or by one third by 2030. Fully achieving the industry reformulation targets is not likely to change this outcome. Our modelling study suggests that the Portuguese industry co-regulation agreement will save lives. However, the overall impact on risk of premature noncommunicable disease deaths is small.

Co-regulation agreements with the food industry, enabled by strong government leadership, with rigorous monitoring might be an effective strategy to change food environments, mitigate risk factors and improve health status. However, we argue that voluntary agreements are insufficient on their own and need to be accompanied by interventions to improve dietary consumption patterns and population health.
